# HYPER‐HIE Phenotype: Postrewarming Systemic Hypertension Following Hypoxic‐Ischemic Encephalopathy—A Case Report and Mechanistic Review

**DOI:** 10.1155/crpe/1742901

**Published:** 2026-07-25

**Authors:** Aimann Surak, Georg M. Schmölzer

**Affiliations:** ^1^ Centre for the Studies of Asphyxia and Resuscitation, Neonatal Research Unit, Royal Alexandra Hospital, Edmonton, Alberta, Canada, royalalex.org; ^2^ Department of Pediatrics, University of Alberta, Edmonton, Alberta, Canada, ualberta.ca

**Keywords:** acute kidney injury, autonomic dysfunction, hypoxic-ischemic encephalopathy, neonatal encephalopathy, perinatal asphyxia, systemic hypertension, systemic vascular resistance, therapeutic hypothermia

## Abstract

**Background:**

Perinatal asphyxia is frequently complicated by cardiovascular instability. Although hypotension and myocardial dysfunction are widely recognized, systemic hypertension after asphyxia has been described in human neonates and may represent a distinct hemodynamic phenotype.

**Case Report:**

We present two infants with hypoxic‐ischemic encephalopathy treated with therapeutic hypothermia who developed systemic hypertension following rewarming and received milrinone as part of hemodynamic management. Blood pressure normalized during the recovery phase while the infants were receiving milrinone; however, the observational nature of these cases precludes conclusions regarding treatment efficacy.

**Review of Literature:**

We provide a narrative review summarizing the available human evidence for postrewarming systemic hypertension and discuss potential mechanisms, implications for phenotype‐directed management, and priorities for future research.

**Conclusion:**

These cases suggest the existence of systemic hypertension emerging after therapeutic hypothermia as a distinct postrewarming hemodynamic phenotype (HYPER‐HIE phenotype), characterized by elevated systemic vascular resistance despite recovering myocardial function. Recognition of this phenotype has important implications for postrewarming cardiovascular surveillance and phenotype‐directed hemodynamic management in neonatal encephalopathy.

## 1. Introduction

Perinatal asphyxia leading to hypoxic‐ischemic encephalopathy (HIE) is a multisystem disorder [1–6], with cardiovascular involvement influencing both acute stability and potentially neurodevelopmental trajectory [7, 8]. Contemporary care emphasizes maintaining adequate cerebral perfusion while avoiding extremes of blood pressure (BP) and oxygenation [9–12]. While hypotension has received substantial attention [8], systemic hypertension following perinatal asphyxia has been described less frequently, suggesting that some infants may develop a vasoconstricted, high‐systemic vascular resistance (SVR) hemodynamic profile [13–15]. We present two infants who were admitted to the neonatal intensive care unit for ongoing care and management of HIE, and subsequently both developed systemic hypertension.

## 2. Case #1

A late preterm female infant was born at 36+3 weeks’ gestation with a birth weight of 3090 g (79th percentile for gestational age), via emergency cesarean section for reduced fetal movements. She was appropriate for gestational age, with no antenatal concerns for fetal growth restriction. The mother was gravida 2, para 1, with negative group B streptococcus screening, protective antenatal serologies, and uneventful pregnancy. The reduced fetal movements were reported for 2 days prior to delivery. At birth, there was thick meconium‐stained amniotic fluid, and the infant was pale, hypotonic, apneic, with no audible heart rate. Resuscitation included positive pressure ventilation followed by cardiopulmonary resuscitation (received two doses of epinephrine), resulting in return of spontaneous circulation (ROSC) at ∼5 min after birth. Apgar scores were 0, 1, and 3 at 1, 5, and 10 min of life, respectively. The infant was passively cooled, followed by therapeutic hypothermia (TH) as criteria were met for HIE. TH continued for a total of 3 days as per standard protocol.

The infant developed meconium aspiration syndrome and severe persistent pulmonary hypertension of the newborn (PPHN), requiring mechanical ventilation, sedation, surfactant administration, and inhaled nitric oxide (iNO) at 20 parts per million. Due to significant cardiovascular instability during the first 48 h of life, the infant required norepinephrine (up to 0.2 μg/kg/min) and dobutamine (up to 10 μg/kg/min). Despite dual vasoactive support, hypotension persisted. Given concern for vasopressor‐resistant hypotension and possible relative adrenal insufficiency, which is commonly encountered in critically ill neonates, a single loading dose of hydrocortisone (2 mg/kg) was administered on Day 2 of life, followed by three additional doses of 1 mg/kg at 6‐h intervals. Hydrocortisone was used as adjunctive therapy to improve hemodynamic stability in the setting of refractory hypotension. Biochemical testing to confirm adrenal insufficiency was not performed prior to treatment; however, empiric hydrocortisone administration in this clinical context is consistent with common neonatal intensive care practice. Gradually, with clinical and echocardiographic improvement, the vasoactive medications and iNO were weaned after 48 h. Clinical seizure activity was noted shortly after admission and treated with loading doses of phenobarbital (20 mg/kg) with no further seizures thereafter. A brain MRI performed on Day 5 showed punctate microhemorrhages and microinfarcts of uncertain clinical significance.

On Day 6 of life, the infant developed persistent systemic hypertension characterized by a maximum systolic BP of 125 mmHg and a maximum diastolic BP of 84 mmHg, all exceeding the 95th percentile for postnatal age [16]. Measurements were obtained invasively via an umbilical arterial catheter. Renal and hepatic Doppler ultrasonography showed normal perfusion and no renal artery thrombosis or structural abnormalities. Urine output remained within the expected range throughout this period, and there was no evidence of clinically significant positive fluid balance or fluid overload. Nephrology consultation recommended diuretic therapy and conservative BP management. Elevated BP readings persisted, and repeated targeted neonatal echocardiography (TnECHO) performed on Day 7 of life demonstrated normal cardiac anatomy, normal biventricular systolic function, no evidence of ventricular hypertrophy, and resolution of the previously identified pulmonary hypertension. Hemodynamic assessment demonstrated markedly elevated estimated SVR (approximately 800 mmHg/l/kg/min; institutional reference range approximately 150–250 mmHg/l/kg/min) despite recovery of ventricular function and resolution of pulmonary hypertension, consistent with a high‐afterload state. There was no evidence of left ventricular outflow tract obstruction, coarctation of the aorta, or other structural cardiac lesions that could account for the elevated BP. Due to persistent hypertension, the TnECHO team recommended milrinone infusion (0.25 mcg/kg/min) as an afterload reduction strategy. BP gradually improved within 24 h, and there was no need for long‐term antihypertensive therapy. Milrinone infusion continued until Day 9 (total of 48 h) and was then discontinued. Table 1 summarizes the timelines of BP changes in relation to TH and rewarming.

**TABLE 1:** Timelines of BP changes in relation to TH and rewarming.

Postnatal day	Clinical phase	Blood pressure status	Associated findings	Interventions	Outcome
Day 0	Birth and Resuscitation	Hypotension	Severe perinatal asphyxia, bradycardia, metabolic derangement	Chest compression and epinephrine	ROSC achieved
Days 0–2	Cooling phase	Hypotension	Severe PPHN, RV dysfunction	Norepinephrine, dobutamine, hydrocortisone, and iNO	Hemodynamic stabilization
Days 3–4	Late cooling–rewarming completed	Normotensive	Improving oxygenation, decreasing inotrope requirement	Gradual weaning of vasoactive medication	Stable during rewarming
Day 6	Postrewarming period	Hypertension	Infant extubated	Nephrology consult, furosemide	Ongoing systemic hypertension
Day 7	Postrewarming period	Hypertension	Normal renal Dopplers, normal urine output	Milrinone infusion (0.25 mcg/kg/min) initiated	Improvement in BP
Days 8–10	Recovery phase	Normotensive	Normotensive	Milrinone discontinued on Day 9; no further antihypertensives required	Stable prior to discharge

Abbreviations: iNO, inhaled nitric oxide; PPHN, persistent pulmonary hypertension of the newborn; ROSC, return of spontaneous circulation; RV, right ventricle/right ventricular.

## 3. Case #2

A term female infant was born at 37+5 weeks’ gestation with a birth weight of 4730 g (99.8th percentile) to a 28‐year‐old gravida 6, para 3 mother. She was large for gestational age, with no antenatal concerns for fetal growth restriction. The pregnancy was complicated by gestational diabetes mellitus that was managed with dietary modification alone and did not require pharmacologic therapy. The mother also had chronic hypertension and superimposed preeclampsia, which were treated with labetalol during pregnancy.

The infant was born via spontaneous vaginal delivery following approximately 1 hour of ruptured membranes. At birth, there was meconium‐stained amniotic fluid, and the infant was pale, hypotonic, apneic, with an audible heart rate > 60 beats per minute. The infant received suctioning of copious meconium‐stained secretions, followed by a brief period of positive pressure ventilation prior to stabilizing on continuous positive airway pressure. Apgar scores were 1, 5, and 5 at 1, 5, and 10 min of life, respectively. Due to persistent respiratory distress and increasing oxygen requirements, the infant was intubated and received surfactant. At 2 h of age, the infant was hypotonic and met criteria for TH, which was provided for 3 days as per standardized protocol.

The respiratory course was significant for severe meconium aspiration syndrome, requiring prolonged mechanical ventilation and administration of two additional doses of surfactant and iNO to treat PPHN. The infant was extubated to CPAP on Day 7 after birth and transitioned to room air by Day 11. The initial echocardiography assessment demonstrated the presence of severe PPHN with right ventricular dysfunction; this necessitated the initiation of iNO (at 20 parts per million) and dobutamine infusion (maximum dose of 10 µg/kg/min). These therapies were gradually weaned over a 3‐day period as pulmonary pressures improved and ventricular function stabilized.

Neurologically, a possible early seizure episode was suspected and treated with phenobarbital (total of 40 mg/kg) and a single loading dose of levetiracetam (60 mg/kg). No further clinical seizures were observed. During TH, continuous electroencephalography demonstrated an abnormal background with slowing and central sharp wave activity, without electrographic seizures. Brain MRI on Day 7 after birth demonstrated a questionable area of restricted diffusion near the cerebellum; however, repeat MRI on Day 14 after birth was normal.

Following completion of TH, the infant developed systemic hypertension characterized by a maximum systolic BP of 117 mmHg and maximum diastolic BP of 79 mmHg, all exceeding the 95th percentile for postnatal age [16]. Measurements were obtained invasively via an umbilical arterial catheter. The systemic hypertension was temporally associated with resolving PPHN and improving ventricular function, as confirmed by follow‐up echocardiography. TnECHO also demonstrated markedly elevated estimated SVR (approximately 750 mmHg/l/kg/min; institutional reference range approximately 150–250 mmHg/l/kg/min), despite improving ventricular performance and resolving pulmonary hypertension.

Milrinone infusion was initiated (0.25 µg/kg/min) for combined management of PPHN and systemic hypertension and was administered for a total of 4 days (between Days 5 and 9). BP normalized with clinical improvement, and milrinone was successfully discontinued on Day 9 without recurrence of hypertension. Table 2 summarizes the temporal relationship between BP changes, TH, and rewarming.

**TABLE 2:** Timelines of BP changes in relation to TH and rewarming.

Postnatal day	Clinical phase	Blood pressure status	Associated findings	Interventions	Outcome
Day 0	Birth and Resuscitation	Normotensive	MAS, respiratory distress	Mechanical ventilation, surfactant	Stabilization
Days 0–3	Cooling phase	Normotensive	Severe PPHN, RV dysfunction	iNO and dobutamine	Improved oxygenation
Days 4–5	Postrewarming period	Rising BP	Resolving PPHN, improving RV function	Milrinone initiated on Day 5	Gradual improvement
Days 6–8	Postrewarming period	Systemic hypertension	Resolving PPHN, improving RV function	Continued milrinone	Gradual improvement
Day 9	Recovery phase	Normotensive	Resolved PPHN, stable cardiac function	Milrinone discontinued	Stable prior to discharge

Abbreviations: iNO, inhaled nitric oxide; MAS, meconium aspiration syndrome; PPHN, persistent pulmonary hypertension of the newborn; RV, right ventricle/right ventricular.

## 4. Hypertension as a Distinct Phenotype: Discussion and Mechanistic Review

Our case report describes two neonates with HIE who developed a postrewarming hypertensive phenotype characterized by systemic hypertension emerging after completion of TH and rewarming, coinciding with recovery of myocardial function and resolution of PPHN. Importantly, echocardiography did not identify structural cardiac abnormalities, ventricular hypertrophy, or significant ventricular dysfunction that would otherwise explain the observed systemic hypertension.

The principal physiological observation in both infants was the emergence of markedly elevated estimated SVR following completion of TH and recovery of myocardial function. We, therefore, hypothesize that persistent elevation in SVR represents the most immediate hemodynamic mechanism underlying the observed postrewarming systemic hypertension in these cases. The temporal transition from early hypotension requiring cardiovascular support to later hypertension despite recovery of ventricular performance suggests a shift from myocardial dysfunction to a predominantly high‐afterload circulation. Although the precise biological drivers of this increase in vascular resistance remain uncertain, the measured hemodynamic profile supports elevated SVR as the most direct explanation for the hypertension observed in these infants. In both infants, TnECHO demonstrated markedly elevated estimated SVR (approximately 800 and 750 mmHg/l/kg/min, respectively; institutional reference range approximately 150–250 mmHg/l/kg/min), despite recovery of ventricular function and resolution of pulmonary hypertension. Although SVR estimation by TnECHO is derived from BP and systemic blood flow measurements and does not represent a direct measure of vascular tone, these findings support increased vascular resistance as a plausible contributor to the observed hypertension. Importantly, the elevated BP in this context may mask impaired systemic blood flow and may have implications for cerebral perfusion during a vulnerable period of neurologic recovery.

Although corticosteroid exposure can contribute to transient BP elevation, hydrocortisone exposure in Case 1 occurred during the early period of cardiovascular instability, whereas systemic hypertension emerged several days later following completion of TH and recovery of myocardial function, making a direct causal relationship less likely.

Other alternative or complementary contributors to systemic hypertension should also be considered. Prior exposure to vasoactive medications may influence vascular tone during recovery; however, in both cases, systemic hypertension emerged after vasoactive support had been discontinued, making an immediate pharmacologic effect less likely. Recovery from severe PPHN and improvement in ventricular function may also unmask a high‐afterload systemic circulation as pulmonary vascular resistance falls and systemic perfusion improves. In Case 2, maternal chronic hypertension, superimposed preeclampsia, and antenatal labetalol exposure represent additional perinatal factors that may have influenced neonatal cardiovascular adaptation. These factors may have contributed to the observed BP phenotype, and the current cases should therefore be interpreted as hypothesis‐generating rather than as demonstrating a single causal pathway.

In contrast to the well‐recognized early hypotension and myocardial dysfunction commonly observed in HIE, these two cases highlight the transition to a hypertensive state during recovery from HIE. Milrinone was selected as a physiology‐based therapeutic strategy because TnECHO suggested a high‐afterload state. BP normalization and clinical improvement occurred following treatment; however, the observational nature of this report precludes conclusions regarding therapeutic efficacy or causality.

We propose the term HYPER‐HIE phenotype (hypertension following hypoxic‐ischemic encephalopathy) to describe this constellation of findings. This proposed entity may reflect a convergence of altered autonomic regulation, dysregulated vascular tone, a catecholamine surge, renal injury, and recovery following HIE and TH [17–21]. In animal model studies, systemic hypertension has been described in the immediate period following ROSC [22]. In our two cases, the elevation in BP was noted upon the completion of TH and rewarming. To our knowledge, this may represent one of the first detailed descriptions of this postrewarming period hypertensive phenotype in human neonates with HIE.

The direct human literature focusing on systemic hypertension following perinatal asphyxia is limited and largely historical, with some reports describing elevated BP in term infants after significant intrapartum hypoxia and related multisystem injury [23, 24]. Contemporary neonatal encephalopathy/HIE cohorts more often report BP profiles, BP variability, vasoactive exposure, and hemodynamic indices during TH, rather than hypertension as the primary endpoint [25, 26]. Additionally, observational data show that vasoactive strategies (e.g., dopamine use during TH) are associated with differing BP profiles, emphasizing that measured BP reflects both illness physiology and treatment effects [27].

Importantly, contemporary studies of neonatal encephalopathy treated with TH have generally focused on hypotension, myocardial dysfunction, vasoactive exposure, BP variability, and systemic blood flow rather than hypertension as a primary endpoint. Consequently, the true incidence and clinical significance of postrewarming systemic hypertension remain uncertain. Furthermore, cardiovascular adaptation during recovery from hypoxic‐ischemic injury is complex, and some increase in BP may represent a physiologic consequence of improving myocardial performance, changing vascular tone, or resolution of pulmonary hypertension rather than a distinct pathologic process. These considerations highlight the need for cautious interpretation of the present observations and underscore the importance of prospective hemodynamic studies during the postrewarming period.

The proposed mechanisms discussed below should be interpreted as potential upstream contributors to the observed increase in SVR rather than independent explanations for the hypertension itself. In the present cases, elevated estimated SVR was the only physiological abnormality directly demonstrated by TnECHO. Consequently, autonomic dysregulation, catecholamine activation, renin–angiotensin–aldosterone system (RAAS) activation, and renal mechanisms should be regarded as biologically plausible pathways that may converge to produce the high‐afterload state, rather than mechanisms established in these patients.

Postrewarming systemic hypertension may be multifactorial, and several biologically plausible mechanisms have been proposed in the literature. Potential contributors include (i) autonomic responses to hypoxia/ischemia, (ii) increased SVR, (iii) endocrine mechanisms, and (iv) renal contributions. These pathways are likely interrelated rather than mutually exclusive and may contribute simultaneously to the observed hemodynamic phenotype. However, none of these pathways were directly evaluated in the present cases and should therefore be regarded as hypothesis‐generating rather than established contributors to the observed hypertension.

Acute hypoxia triggers sympathetic outflow and a catecholamine surge, producing peripheral vasoconstriction and potentially elevated BP. Animal model studies demonstrate that acute hypoxia upregulates mRNA expression of the adrenal catecholamine synthetic enzymes (tyrosine hydroxylase and phenylethanolamine N‐methyltransferase) [28–31].

A key clinical pitfall is that elevated BP can coexist with and mask a state of compromised systemic blood flow when SVR is high and myocardial performance is impaired, a common finding following asphyxia. Neonates with HIE may exhibit apparently normal BP despite compromised systemic blood flow, owing to compensatory peripheral vasoconstriction, which may be exacerbated by TH. Multimodal monitoring studies during TH and rewarming demonstrate that cardiac output and SVR‐related indices can change independently of BP, reinforcing that BP does not always reflect perfusion adequacy [24, 32–34]. Bedside indicators of systemic blood flow such as heart rate, BP, and lactate are limited in their ability to accurately reflect circulatory adequacy [35–37]. Although markers such as lactic acidosis may reflect the severity of the initial insult, they do not reliably correlate with real‐time cardiac output [38].

The RAAS plays a critical role in the response to asphyxia. It is a key regulator of SVR, intravascular volume, and arterial BP, and is particularly active during fetal and early neonatal life, when baseline renin and angiotensin II levels are physiologically elevated [39, 40]. In the setting of perinatal asphyxia, reduced renal perfusion, hypoxia, and sympathetic activation lead to acute upregulation of RAAS, resulting in angiotensin II–mediated vasoconstriction and aldosterone‐driven sodium and water retention aimed at maintaining systemic BP [41–44]. While initially adaptive, excessive or sustained RAAS activation may become maladaptive, contributing to increased afterload, impaired systemic blood flow, and end‐organ injury, particularly in the presence of evolving renal dysfunction [45–47].

Renal injury is common following perinatal asphyxia and may contribute to dysregulation of BP through disturbances in sodium and water handling and activation of neurohumoral pathways, including the RAAS mentioned above [48–50]. Contemporary reviews and outcome studies consistently demonstrate a high incidence of acute kidney injury in neonates with HIE, including those treated with TH, and highlight important implications for both short‐term hemodynamic instability and longer term renal outcomes [45, 51–53]. The pathophysiologic mechanisms of renal injury in HIE are likely related to the combined effects of hypoxic‐ischemic injury, altered renal perfusion, and hypothermia‐related changes in renal physiology [53]. Interpretation of additional renal parameters, such as urine output, remains challenging in this population, as oliguria may reflect the primary hypoxic insult, renal hypoperfusion, evolving kidney injury, or the direct physiologic effects of hypothermia itself, rather than isolated intravascular volume status [6]. In the present cases, urine output remained within expected limits and clinically significant fluid overload was not observed, suggesting that volume excess alone was unlikely to account for the observed hypertension.

Nevertheless, renal and neurohumoral mechanisms may still contribute to altered vascular tone even in the absence of overt fluid overload. In neonates with HIE, the interaction between asphyxia‐induced RAAS activation, TH, and renal injury may further amplify SVR, predisposing to postrewarming systemic hypertension despite improving myocardial function, supporting a potential mechanistic role for RAAS dysregulation in postrewarming BP instability [12, 54].

The delayed emergence of hypertension following discontinuation of vasoactive medications suggests that the observed phenotype was unlikely to represent a direct pharmacologic effect and instead may reflect evolving postasphyxial cardiovascular adaptation during the recovery phase after TH.

### 4.1. Limitations

This report has several limitations. The proposed mechanisms underlying postrewarming systemic hypertension were not directly measured in either infant. Specifically, catecholamine concentrations, RAAS activity, SVR, and quantitative measures of systemic blood flow were not assessed. Although TnECHO demonstrated markedly elevated estimated SVR in both infants, these estimates were derived from BP and systemic blood flow measurements and do not represent direct measurements of vascular tone. Serial measurements of estimated SVR throughout TH and following discontinuation of milrinone were not available. Consequently, we cannot determine whether elevated SVR was already present during TH or developed predominantly during the postrewarming period. Prospective serial hemodynamic assessment will be important to better characterize the evolution of this proposed phenotype. In addition, neither infant demonstrated overt renal dysfunction or clinically significant fluid overload at the time hypertension was observed. Furthermore, the prevalence, reproducibility, and long‐term clinical implications of this proposed phenotype remain unknown and require evaluation in larger prospective studies.

Similarly, the observed temporal association between milrinone administration and BP normalization should not be interpreted as evidence of treatment efficacy, as spontaneous recovery and other unmeasured factors may have contributed to the clinical course. Consequently, the mechanistic explanations presented should be considered hypothesis‐generating and intended to provide a conceptual framework for future investigation rather than to represent definitive causal pathways.

## 5. Conclusion and Future Directions

Recognition of a potential HYPER‐HIE phenotype may have important clinical implications, as unanticipated systemic hypertension emerging after rewarming may disrupt cerebral perfusion and may contribute to secondary brain injury. In the era of TH, accumulating evidence from multimodal monitoring indicates that BP must be interpreted in conjunction with systemic blood flow, vascular resistance, renal function, and cerebral autoregulatory vulnerability, rather than as an isolated parameter. Our observations highlight the potential need for continued cardiovascular surveillance beyond the TH period and justify a phenotype‐directed, physiology‐guided approach to hemodynamic management, incorporating TnECHO and phase‐specific monitoring, to optimize cardiovascular care and neuroprotection in infants with HIE.

Although both infants improved following milrinone therapy, the present report is not designed to determine treatment efficacy, and prospective studies are required to evaluate optimal management strategies for this proposed phenotype.

**Nomenclature**
BP: Blood pressure
CPAP: Continuous positive airway pressure
HIE: Hypoxic‐ischemic encephalopathy
iNO: Inhaled nitric oxide
MAS: Meconium aspiration syndrome
NICU: Neonatal intensive care unit
PPHN: Persistent pulmonary hypertension of the newborn
RAAS: Renin–angiotensin–aldosterone system
ROSC: Return of spontaneous circulation
SVR: Systemic vascular resistance
TH: Therapeutic hypothermia
TnECHO: Targeted neonatal echocardiography

## Author Contributions

Aimann Surak and Georg M. Schmölzer contributed to the conception and design of the study.

Aimann Surak drafted the initial manuscript.

Georg M. Schmölzer critically revised the manuscript for important intellectual content.

## Funding

The authors have nothing to report.

## Disclosure

All authors read and approved the final manuscript.

## Ethics Statement

The authors have nothing to report.

## Consent

Parental consent was available upon request.

## Conflicts of Interest

The authors declare no conflicts of interest.

## Data Availability

Data sharing is not applicable to this article as no datasets were generated or analyzed during the current study.
